# Antimicrobial Peptides: The Game-Changer in the Epic Battle Against Multidrug-Resistant Bacteria

**DOI:** 10.3390/ph17111555

**Published:** 2024-11-20

**Authors:** Helal F. Hetta, Nizar Sirag, Shumukh M. Alsharif, Ahmad A. Alharbi, Tala T. Alkindy, Alanoud Alkhamali, Abdullah S. Albalawi, Yasmin N. Ramadan, Zainab I. Rashed, Fawaz E. Alanazi

**Affiliations:** 1 Division of Microbiology, Immunology and Biotechnology, Department of Natural Products and Alternative Medicine, Faculty of Pharmacy, University of Tabuk, Tabuk 71491, Saudi Arabia; hhussen@ut.edu.sa (H.F.H.); aam_alharbi@ut.edu.sa (A.A.A.); talkendy@ut.edu.sa (T.T.A.); 2 Division of Pharmacognosy, Department of Natural Products and Alternative Medicine, Faculty of Pharmacy, University of Tabuk, Tabuk 71491, Saudi Arabia; nizarsirag@gmail.com; 3 Department of Pharmaceutical Chemistry, Faculty of Pharmacy, University of Tabuk, Tabuk 71491, Saudi Arabia; aalkhamali@ut.edu.sa (A.A.); abs_albalawi@ut.edu.sa (A.S.A.); 4 Department of Microbiology and Immunology, Faculty of Pharmacy, Assiut University, Assiut 71515, Egypt; yasmine_mohamed@pharm.aun.edu.eg (Y.N.R.); zeinab_rashed@pharm.aun.edu.eg (Z.I.R.); 5 Department of Pharmacology and Toxicology, Faculty of Pharmacy, University of Tabuk, Tabuk 71491, Saudi Arabia

**Keywords:** antimicrobial peptides (AMPs), antibiotic resistance, MDR

## Abstract

The rapid progress of antibiotic resistance among bacteria has prompted serious medical concerns regarding how to manage multidrug-resistant (MDR) bacterial infections. One emerging strategy to combat antibiotic resistance is the use of antimicrobial peptides (AMPs), which are amino acid chains that act as broad-spectrum antimicrobial molecules and are essential parts of the innate immune system in mammals, fungi, and plants. AMPs have unique antibacterial mechanisms that offer benefits over conventional antibiotics in combating drug-resistant bacterial infections. Currently, scientists have conducted multiple studies on AMPs for combating drug-resistant bacterial infections and found that AMPs are a promising alternative to conventional antibiotics. On the other hand, bacteria can develop several tactics to resist and bypass the effect of AMPs. Therefore, it is like a battle between the bacterial community and the AMPs, but who will win? This review provides thorough insights into the development of antibiotic resistance as well as detailed information about AMPs in terms of their history and classification. Furthermore, it addresses the unique antibacterial mechanisms of action of AMPs, how bacteria resist these mechanisms, and how to ensure AMPs win this battle. Finally, it provides updated information about FDA-approved AMPs and those that were still in clinical trials. This review provides vital information for researchers for the development and therapeutic application of novel AMPs for drug-resistant bacterial infections.

## 1. Introduction

Resistance to antibiotics represents a severe danger to global health, resulting in roughly 1.27 million deaths annually and over 5 million fatalities in 2019. Antibiotic resistance affects more than 2.8 million diseases in the US annually. According to the CDC’s 2019 Antibiotic Resistance (AR) Threats Report, over 35,000 individuals have died as a result [1]. It is estimated that in less than 30 years, AR could become more lethal than cancer, and that it will kill 10 million people per year by 2050 [2].

The rapid emergence of antibiotic resistance is strongly connected to the overuse of antibiotics, their widespread use in agriculture, and the lack of innovative antibiotics [3,4,5]. Since the development of bacterial drug resistance became more frequent, early detection and treatment of drug-resistant bacterial illnesses has become extremely critical [6,7]. Although antibiotics were formerly capable of treating the majority of bacterial illnesses, the advent of AR has reduced the efficacy of conventional antibiotics [8]. Thus, it is necessary to identify effective alternatives. Antimicrobial peptides (AMPs) are now considered a potential antibacterial approach due to their broad-spectrum action, significant effect on Gram-negative bacteria, and low drug resistance [9]. In addition to directly fighting germs, AMPs also have immunomodulatory characteristics [10]. This makes them particularly attractive substances for the development of new medicines. Clinical trials are being conducted on numerous AMPs, and there are encouraging instances of AMPs that have previously been released on the market [11], justifying the introduction of innovative AMP-based medications in several therapeutic areas [12].

AMPs are superior to traditional antibiotics for several reasons. For instance: (1) They are simpler to manufacture because they frequently comprise short sequences of amino acids [13]; (2) They are efficient against conventional antibiotic-resistant strains, potent with quick germ-killing capacity [14] and low bactericidal concentration, and even have synergistic actions with conventional antibiotics [15,16,17,18,19]; (3) They have no impact on the gut microbiome, which conventional antibiotics frequently disturb [20]; and (4) They have good heat stability and water solubility [21,22]. Due to these benefits, various AMPs, including nisin, gramicidin, polymyxins, daptomycin, and melittin, are currently used in medicine [19].

## 2. Development of Antibiotic Resistance

**Figure 1:**
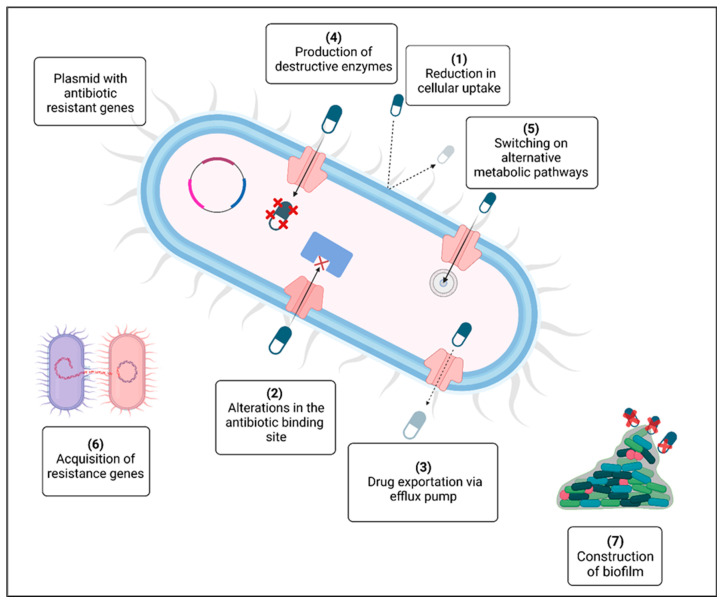
Possible mechanisms for the development of antibiotic resistance. A variety of mechanisms contribute to its development: (1) drug breakdown through the production of an inactivating enzyme; (2) reduction in antibiotic uptake; (3) modification of a common metabolic pathway; (4) drug extrusion from the cell by an efflux pump; (5) modification of the antibiotic target; (6) presence of a plasmid containing multiple resistance genes; (7) sharing of resistance genes among bacteria; and (8) formation of a protective extracellular polymeric matrix. The red cross (X) represents destructive enzymes [27]. Created with BioRender.

Bacteria are dynamic living things that adapt throughout time. Their main objective is to grow, endure, and disperse as widely as possible [23]. Bacteria subsequently evolve in ways that ensure their continued existence and accommodate their surroundings. Genetic changes may occur if any substance, such as an antibiotic, stops them from developing, making them more resistant to the treatments and allowing their persistence [24,25]. Bacteria exhibit several molecular pathways via which they can render antibiotics ineffective and useless [26,27] (Figure 1).

- Reduction in cellular uptake: Antibiotic entry can be lowered by structural design alterations to the surface casing. In Gram-negative bacteria, for example, a modification or decline in the number of porins, or mutation in the porin gene, may be the source of resistance [28,29].
- Alterations and modifications to the antibiotic binding site: methylation of an adenine residue in the peptidyl-transferase of rRNA 23S decreases the affinity of the enzyme for the antibiotic without affecting protein synthesis, and this participates in the development of erythromycin resistance [30,31]. Penicillin-binding proteins (PBPs) modified by methicillin-resistant *Staphylococcus aureus* (MRSA) provide yet another crucial instance [32].
- Drug exportation via an efflux pump: antibiotics that bacterial cells have absorbed can be expelled via an energy-dependent drug efflux pump. Frequently, antibiotic-resistant bacteria possess upregulated efflux pumps [29,33,34].
- Secretion of destructive enzymes: When particular enzymes generated by bacteria selectively inactivate the antibiotic, it loses its biological function. For example, this happens when β -lactamases break down β-lactam antibiotics. Some bacteria produce extended-spectrum β-lactamases (ESBLs), which share a comparable inactivating property and make them difficult to eradicate. Additionally, certain antibiotics may lose their effectiveness due to other enzymes, such as acetyltransferase, phosphotransferase, and adenyl transferase [35,36,37,38,39].
- Switching on alternative metabolic pathways: as an illustration, consider the sulfonamides scenario. Sulfonamide-exposed bacteria continue to generate folic acid via an alternative metabolic route [40].
- Acquisition of resistance genes: resistance genes spread throughout bacterial species because of microbial cooperation and genetic data exchange [3,41,42,43,44,45,46,47,48,49,50,51,52,53].
- Construction of biofilm: the biofilm is mainly composed of a mesh of extracellular polymeric matrix. Many antibiotics, like aminoglycosides, cannot penetrate this fortified matrix because of electrostatic repulsion force [41,54,55,56,57,58,59,60].
- Being in a dormant state: persister cells, a physiologically dormant subset of bacteria, are more resistant to antibiotics than active bacteria [61].

## 3. Background of AMPs

AMPs are chemicals that have been preserved throughout evolution and are present in prokaryotes as well as humans. In 1922, Alexander Fleming found the first human antimicrobial protein, lysozyme, in nasal mucus. This observation was eclipsed in 1928 by Fleming’s discovery of penicillin and the subsequent development of its therapeutic uses in the 1940s, which earned him the 1945 Nobel Prize in Medicine. Thus, the therapeutic potential of endogenous AMPs like lysozyme was neglected while the “Golden Age of Antibiotics” began in the 1940s. In the 1960s, the discovery of multidrug-resistant microbial infections prompted research into the potential of AMPs as host defense agents [62]. 

AMPs have been produced by all known living organisms. The discovery of AMPs probably began with plants, and in the 1960s, lactoferrin from milk and brombinin from frogs were both described [62].

A pioneering study from 1981 described how bacteria were injected into *Cecropia moths* to yield a strong AMP. Another significant development came about in 1987 when scientists extracted and studied cationic AMPs from the African clawed frog *Xenopus laevis*. The crucial role of AMPs in insect host defense was originally demonstrated in the mid-1990s when research revealed that genetic excision of AMP synthesis rendered the fruit fly susceptible to a severe fungal infection [62].

Currently, the AMP Database contains more than 3569 AMPs from six different life kingdoms (https://aps.unmc.edu/home (accessed on 5 April 2024)).

## 4. Classification of AMPS

**Figure 2:**
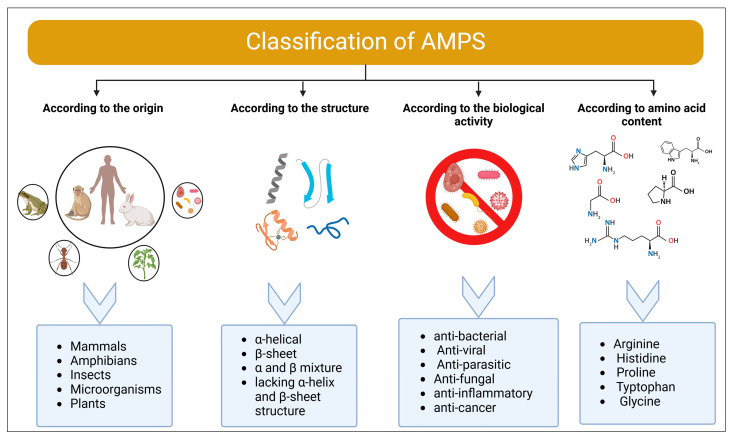
Classifications of AMPs according to origin, structure, activity, and amino acid content. Created with BioRender.

AMPs are a broad set of molecules with distinct chemical structures and amino acid compositions. The AMP Database currently categorizes AMPs based on a variety of characteristics [63]. In general, AMPs are classified based on several main criteria; source, structure, activity, and amino acid content [64] (Figure 2). 

### 4.1. Classification According to the Origin

According to statistical information in APD3, the origins of AMPs can be classified as mammals (of which human host defense peptides make up a significant portion), amphibians, plants, microbes, and insects [65].

#### 4.1.1. AMPs Derived from Mammals

Humans, sheep, cattle, and other vertebrates all produce mammalian AMPs. The two primary families of AMPs are cathelicidins and defensins. Based on the location of the disulfide bond, defensins can be classified as α-, β-, and θ-defensins [66]. Human host defense peptides (HDPs) can protect the body from potential pathogens, but they do so in a several ways depending on the stage of development. While cathelicidin LL-37, an AMP produced by our body, is generally observed in the skin of babies, human beta-defensin 2 (hBD-2) is frequently produced in older people, not youth [67]. HDPs have been identified in a variety of human parts, including the skin, ears, eyes, mouth, urethra, gut, lungs, and respiratory system. Furthermore, AMPs found in human breast milk are essential for lactation because they can reduce the mortality and morbidity of nursing neonates [68]. The discovery of casein 201 in colostrum is remarkable because it demonstrates variable levels in term and preterm human colostrum [69]. Milk products are a large reservoir of AMPs, which are created when milk is hydrolyzed enzymatically. Many AMPs have been found, such as casein, lactoferrin, β-lactoglobulin, and α-lactalbumin fractions; the most well-known of them is lactoferricin B (LfcinB) [70].

Moreover, HDPs, such as cathelicidins and defensins, influence immunological control, apoptosis, and wound healing besides having antimicrobial properties [71].

#### 4.1.2. AMPs Derived from Amphibians

Amphibian AMPs are essential to protect amphibians from infections that are contributing to the global decline in amphibian population [72]. Frogs are the source of the bulk of amphibian AMPs, and magainin is the most popular AMP produced by frogs. It was reported that the skin secretions of frogs belonging to the genera *Xenopus, Silurana, Hymenochirus*, and *Pseudhymenochirus* in the *Pipidae* family are rich in AMPs [73]. Additionally, Cancrin was reportedly the first AMP to be isolated from the marine amphibian *Rana cancrivora* [74].

#### 4.1.3. AMPs Derived from Insects

AMPs are largely produced by the blood cells and fat bodies of insects, which is one of the key factors in their high level of survival adaption [75]. The most popular class of AMPs from insects is called cecropin, which is found in *Drosophila*, bees, and guppy silkworms [76]. Jellein, a kind of AMP generated by bee royal jelly, was shown to be effective against a wide range of bacteria and fungi, and its conjugated molecule with lauric acid can stop the parasite *Leishmania major* [77].

#### 4.1.4. AMPs Derived from Microorganisms

There is a chance of acquiring antimicrobial peptides from microbes such as bacteria and fungi. Notable examples of such peptides are gramicidin and nisin from *Brevibacillus brevis* and *Lactococcus lactis*, respectively [78].

#### 4.1.5. AMPs Derived from Plants

Plant barrier defense systems are made up of AMPs that are produced by plants. They come in a wide variety of genera and species and can be obtained from different plant components such as roots, seeds, flowers, leaves, and stems. Moreover, they participate in phytopathogen activities and have antibacterial defenses against a variety of microorganisms, including those that are harmful to humans [79].

Plant AMPs are categorized according to genetic sequences that are comparable to each other, cysteine-rich motifs, and disulfide connections that reveal information about the folding of their tertiary structure. These families include thionins, plant defensins, snakins, proteins of the knottin and hevein types, α- hairpinin families, etc. More information regarding the AMPs from plants is set out in Table 1 [80].

### 4.2. Classification According to Structure

AMPs are classified into four classes, depending on their secondary structure: α-helical, β-sheet, α and β mixture, and lacking α-helix and β-sheet structure (extended peptide) [83]. AMPs with an α-helical structure have a unique and functionally important arrangement of amino acid chains in a helical spiral shape that gives them particular characteristics that affect how well they interact with microbial membranes and how effective they are as antimicrobial agents overall [84]. AMPs with β-sheet structures fold into sheets of amino acid chains. This structural motif is frequently stabilized by intramolecular disulfide bonds, which contribute to the peptides’ overall stability and activity [64]. AMPs with an αβ-mixture structure have a hybrid structure of both α-helical and β-sheet components in their peptide chains. These peptides frequently exhibit amphipathicity, meaning that they include both hydrophobic and hydrophilic amino acid sequences [85]. Extended/random coil antimicrobial peptides are distinguished from the usual α-helical or β-sheet structures by their elongated or looped conformations. This structural variety leads to their capacity to interact with bacteria membranes and exhibit antimicrobial actions [86]. 

Crucially, each AMP’s primary amino acid component determines its net charge and, consequently, its mode of interaction with the bacterium. For example, anionic AMPs are composed of 5 to 70 amino acid residues with a net charge ranging from −1 to −8. Their structural characteristics include α-helical peptides from some amphibians and cyclic cystine knots [87]. They appear to use metal ions and the negatively charged components found in the bacterial membrane to generate salt bridges, thus interacting with bacteria [88], which is comparable to the charge-neutralization features of larger proenzymes [89]. The first known anionic AMP containing 5–7 aspartate residues, ovine pulmonary surfactant associated anion peptide (SAAP), demonstrated antibacterial action against the ovine pathogen *Mannheimia haemolytica* when zinc ions were present [90].

Cationic α-helical AMPs are another example. These peptides are small (less than 40 amino acids) and often have a net charge ranging from +2 to +9 with an amidated C-terminus [91]. In water, these peptides are disordered. However, when exposed to trifluoroethanol, SDS micelles, phospholipid vesicles, or liposomes, they partially or totally adopt an α-helical shape [92]. Furthermore, these AMPs often have more than 50% hydrophobic amino acids, which allow for the development of amphiphilic structures during interactions with target bacterial cells [93]. Cathelicidins are often amphiphilic α-helical AMPs [94], such as LL-37, which have been extensively investigated. LL-37 is the only human cathelicidin of an active fragment released from hCAP18 by serine protease 3 in neutrophils, with a net charge of +6 at neutral pH [95,96]. The efficiency of structural transformation from disordered into an α-helical structure is directly related to peptide concentrations. Furthermore, LL-37’s antibacterial action against both Gram-positive and Gram-negative bacteria is associated with the degree of the α-helix [97]. 

The cationic β-sheet AMPs are the third example. Typically, the peptides have two to eight cysteine residues that form one to four intramolecular disulfide bond pairs [98]. The stability of these peptides’ structures and biological activities depends on their disulfide bonds. For instance, they become inactive when cysteines are substituted by acidic amino acids, but remain active when mutated to hydrophobic amino acids (except alanine and leucine) [99]. Defensins make up the majority of the β-sheet AMPs [98]. In the case of α-defensins, near the amino terminus, they form a three-stranded chain by hydrogen bonding with the β-hairpin, and a cyclic structure by pairing cysteine with disulfide bonds [100]. The bactericidal action of amphipathic α-defensins is dependent on the hydrophobic and positively charged amino acids that interact with phosphatidyl chains to destroy bacterial membranes [101]. Furthermore, cationic α-defensin residues may engage with negatively charged bacterial surface materials before hydrophobic residues contact with the membranes, thereby mediating membrane breakdown and bacterial death [102]. On the other hand, some β-defensins are composed of both α-helix and β-sheet structures. For example, the insect defensin A contains an α-helix with 11 amino acid residues in the middle (residues 14–24), and its N-terminal β-hairpin is parallel to the α-helix. The first 13 amino acid residues create a cyclic structure [103]. Antibacterial and antiparasitic actions are primarily controlled by the N-terminal domain of the chicken Gga-AvBD11 and augmented by its C-terminal domain [104]. θ-defensins are end-to-end cyclized tetracyclic peptides with three disulfide bridges connecting antiparallel β-sheets. The antibacterial characteristics of θ-defensins are bolstered by their cyclic structure, which keeps them active at high salt concentrations. The loss of this structure results in a decrease in microbicidal activity [105,106]. 

### 4.3. Classification According to Biological Activity

Another criterion with which to classify AMPs is based on their biological activity, and depending on the specific types of microorganisms they target, they can be antibacterial, antiviral, antiparasitic, antifungal, anti-inflammatory, anti-cancer, anti-fibrotic, anti-HIV, etc. According to statistical data provided by APD3, antibacterial AMPs form the largest proportion, accounting for 55 %, while antifungal peptides form 29 %. Smaller fractions (around 2–5 %) of the peptides consist of antiviral, antiparasitic, anti-cancer, anti-HIV, or other functions [107]. 

### 4.4. Classification According to Amino Acid Content

AMPs are highly diverse; however, they can be classified based on features such as amino acid content, charge, and hydrophobicity. AMPs are amino acid-rich peptides containing arginine, histidine, proline, tryptophan, and glycine. The term “rich” refers to the existence of specific amino acids in the sequence with a minimum frequency of 25% [108]. AMPs are categorized as cationic (88%), neutral (6%), and anionic peptides (6%) based on their net charge [109]. The majority of AMPs are amphipathic, although, in rare circumstances, AMPs are entirely made up of hydrophobic or hydrophilic amino acids [110]. 

## 5. Mechanisms of AMPs for Combating MDR Bacteria 

Understanding AMPs and their mechanism of action will provide information on how to create the next generation of synthetic AMPs that are effective [111,112].

**Figure 3:**
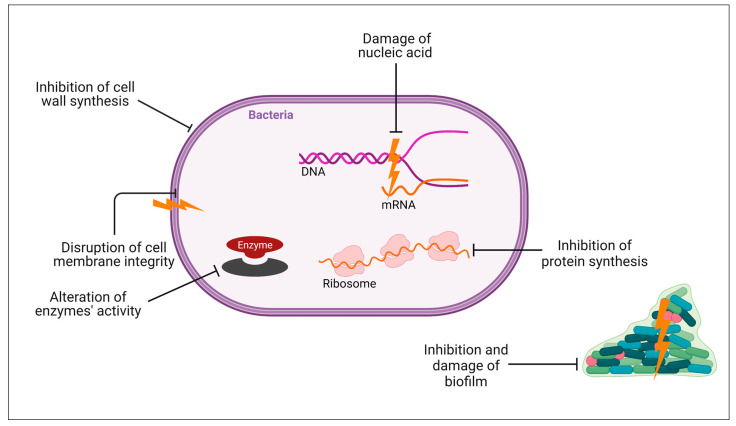
Different antibacterial mechanisms of AMPs. AMPs can exhibit different antibacterial mechanisms, including cell membrane damage, intracellular bactericidal mechanism, damage to organelles that results in DNA fragmentation, inhibition of enzyme activity, as well as inhibition and damage of biofilm. Created with BioRender.

**Figure 4:**
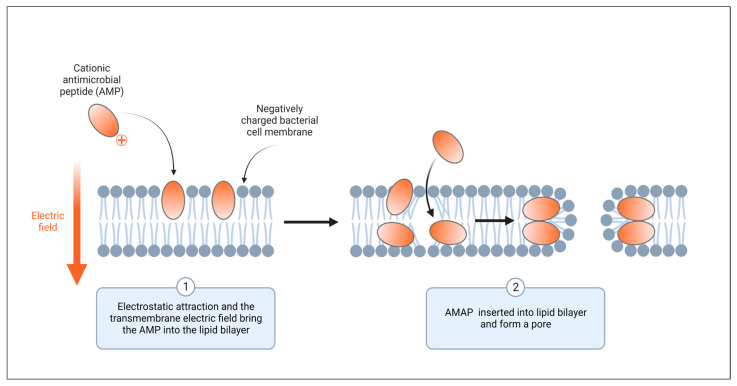
Antimicrobial peptides disrupt membrane integrity. The presence of a positive charge on AMPs results in electrostatic attraction with bacterial cell membrane due to the negative charge present. This electrostatic attraction ends with the insertion of the AMP into the lipid bilayer and forming pores. Created with BioRender.

Currently, there are numerous hypothetical mechanisms of action for these peptides, including cell membrane damage, intracellular bactericidal mechanism, inhibition of macromolecule synthesis, damage to organelles that results in DNA fragmentation, inhibition of enzyme activity, and antimicrobial effect through involvement in immune regulation [113,114] (Figure 3). The interaction between cationic AMPs and cell membranes is believed to be one of the potential and recognized mechanisms (Figure 4).

### 5.1. AMP-Bacterial Membrane Interaction

Numerous AMPs have an immediate and robust antibacterial impact by affecting the integrity of the microbial cell membrane and/or penetrating the membrane to engage intracellular targets [115]. Membrane interaction is an essential step in an AMP’s direct antibacterial action, whether the membrane is directly targeted or translocated to an intracellular target [116,117,118,119]. Electrostatic force interactions that occur between the cationic antimicrobial peptides and the negative charge on the bacterial membrane greatly affect how peptides interact with the microbial membrane [11,118,120,121] (Figure 4).

**Figure 5:**
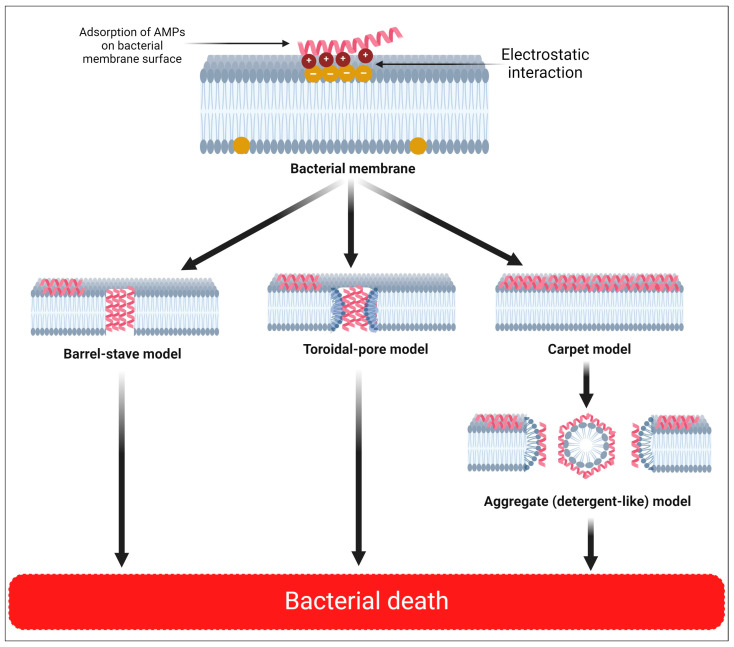
Models of AMPs antibacterial defense systems. The interaction of negatively charged bacterial cell membranes with AMPs increases the permeability and cell lysis of the membrane, or the release of cytoplasmic components, all of which cause cell death as part of their direct bactericidal mechanism. The creation of membrane pores can be modeled using the barrel-stave, toroidal-pore, carpet, and aggregate models, respectively. The hydrophobic sections of AMPs that pass through the phospholipid membrane mix with the hydrophobic regions in the inner area of the phospholipid bilayer, leaving the hydrophilic regions exposed. Another bactericidal strategy involves antimicrobial peptides penetrating the cytoplasm and interacting with intracellular elements, such as blocking protein folding, enzyme activity, DNA, RNA, and protein production. Created with BioRender.

Several possible membrane-cavity formation models have been put forth, including the barrel-stave, toroidal-pore, carpet, and aggregate models (Figure 5).

#### 5.1.1. The Barrel-Stave Model

Higher peptide binding causes aggregation and conformational change, which thins the membrane and moves the local phospholipid head groups [122]. While the hydrophobic sections of the α-helical peptides and β-sheet peptides are adjacent to the hydrophobic groups of the membrane phospholipid, the hydrophilic sections of the peptide helixes face inwards during the entry into the phospholipid bilayer. The central lumen is subsequently formed by the parallel arrangement of many helical molecules [123].

#### 5.1.2. The Toroidal-Pore Model

The toroidal-pore model is like the barrel-stave model, but differs in that the peptide helix enters membranes and binds to lipids to produce the toroidal-pore complexes. Peptides and lipid head groups anchored within the hydrophobic center of the lipid can move about because high concentrations of locally deposited AMPs cause lipid molecules to move freely [122].

#### 5.1.3. The Carpet Model

While the anionic membrane and AMP electrostatic action are required, the formation of micelles and destruction of the microbial membrane require high AMP concentrations [123]. Clusters of AMPs coat the membrane once the peptide concentration exceeds the threshold, breaking it like a surfactant. The hydrophobic core of the membrane is not penetrated by the peptides, and the channel is not formed. This activity is potent enough to lyse the cell membrane, resulting in cell death [124]. 

#### 5.1.4. The Aggregate Model 

A peptide-lipid complex micelle is formed by attaching AMPs to the negatively charged bacterial cell membrane [125]. As opposed to the carpet model, AMPs, lipids, and water create channels that let ions and cellular contents escape, ultimately leading to cell death. The movement of AMPs into the cytoplasm, where they can function, may also be facilitated by such channels. This procedure explains why, in addition to the cytoplasmic membrane, which is the major target of AMPs, they can also act on intracellular molecules [126].

The mechanisms of action of anionic AMPs remain a mystery. It has been proposed that the antibacterial efficacy of maximin H5 against *Staphylococcus aureus* (*S. aureus*) may be influenced by membrane breakdown [127]. The aspartic acid residues in maximin H5 significantly contribute to the structure of the N-terminal α-helical peptide, which mediates interaction with microorganisms due to its distance from the membrane surface. The hydrogen bonds formed by the amidation of the C- and N-terminal regions of the peptide are crucial for the stability of the structure of α-helical peptides [124]. Additionally, low pH appears to support the stability of the α-helix structure of maximin H5 and promotes the death of *S. aureus* through a process resembling the “Carpet” mechanism [127]. Moreover, it has been demonstrated that the anionic AMP Xlasp-p1 has potent broad-spectrum antibacterial activity against both Gram-positive and Gram-negative bacteria by rupturing cell membranes and encouraging the efflux of cytoplasmic contents [128].

### 5.2. Intracellular Mode of Action

There is mounting evidence that AMPs have additional processes in addition to membrane penetration and hole creation. 

#### 5.2.1. AMPs Acting on Nucleic Acids 

AMPs have the potential to kill bacteria by specifically targeting their nucleic acids. An AMP called buforin II, which comprises 21 amino acids, is antimicrobial to a variety of bacteria [129]. It shares the same sequence as a region of the protein known as histone H2A, which directly interacts with nucleic acids [129]. Previous research has demonstrated that buforin II bonded to DNA and RNA and entered lipid vesicles in vitro without altering membrane permeability [129]. 

Like that, indolicidin entered the bacterial membrane, preventing DNA synthesis when there is no bacterial cell lysis [129]. 

An anionic antimicrobial peptide called Peptide-P2 that was found in the skin of *Xenopus laevis* prevented the growth of bacteria by rupturing their cell membranes and interacting with their genomic DNA [130]. 

#### 5.2.2. AMPs Influence Protein Synthesis

PR-39, a high proline and arginine AMP derived from pigs’ small intestines, was discovered to rapidly enter *E. coli*’s outer membrane [131]. As soon as PR-39 enters the cytoplasm, it stops the synthesis of proteins and triggers the breakdown of proteins required for DNA synthesis, which in turn limits DNA synthesis. Typically, the proline-enriched AMPs attach to ribosomes and inhibit the synthesis of proteins [132]. For instance, apidaecin-type peptide prevents the construction of the ribosome’s 50S large subunit while oncocin-type peptide limits mRNA translation by binding 70S ribosome export [133]. It has been demonstrated that the peptide api137, generated from apidaecin, binds to *E. coli* ribosomes and inhibits the release of either RF1 or RF2, which stops translation [134]. 

#### 5.2.3. AMPs Influence Enzymes’ Activity

It was discovered that the post-translationally modified peptide microcin J25, which is produced by ribosomes, attaches to the secondary channel of RNA polymerase, and inhibits trigger-loop folding, which is necessary for the efficient catalysis of RNA polymerase. As a result, it suppresses RNA polymerase activity by blocking material availability through this channel [135]. 

Studies revealed that LL-37 would have a strong action on *E. coli* by inhibiting the action of palmitoyl transferase PagP, which is present in the outer membrane of Gram-negative bacteria and restores membrane permeability by activating lipids acylation [136]. 

Other studies indicate that the NP-6 AMP from Sichuan pepper seeds significantly and dose-dependently reduced the β-galactosidase activity of *E. coli* [137].

#### 5.2.4. AMPs Influencing Cell Wall Synthesis

Teixobactin strongly interfered with the formation of teichoic acid (lipid III) and peptidoglycan (lipid I and II) metabolism in bacterial cell walls and enhanced the killing of resistant organisms such as MRSA through the disruption of cell walls [138,139].

### 5.3. Inhibition and Damage of Biofilm

Pathogens can resist the effects of traditional antimicrobials more readily when they are in the form of biofilms. Although they are completely susceptible in planktonic settings, microbes in biofilms can tolerate large doses of antimicrobials, particularly via transcriptional regulatory mechanisms [140].

While the precise method by which AMPs affect biofilms is still unknown, it is recognized that they can prevent the development of biofilms at different stages by inhibiting the adhesion of free cells to the biofilm or modifying the shape of free cells [141].

It has already been proven that certain AMPs can stop bacteria from adhering to surfaces by inhibiting motilities like “swarming” or “swimming”. They can also inhibit “twitching” motility and promote the breakdown of biofilms [142]. Biofilm production is the primary cause of infection for many bacteria, including MDR-*P. aeruginosa*. According to relevant research, AMP P5 produces an antibacterial effect by disrupting the biofilm structure and promoting the death of carbapenem-resistant *P. aeruginosa* [143]. Additionally, quorum-sensing genes, known to be important for biofilm development, bacterial organization, and communication within the biofilm, can be downregulated by AMPs [140]. In light of this, it has been shown that LL-37 cathelicidin particularly targets the quorum-sensing systems that regulate the production of *P. aeruginosa* biofilms [144].

AMPs can effectively eliminate MDR-*S. aureus* infection by disturbing the structure of the biofilm and subsequent deep penetration. Accordingly, Mohamed et al. [145] synthesized two novel short-chain peptides (WR12 and D-IK8) and evaluated their activities against MRSA. Amazingly, the authors discovered that these short peptides significantly inhibit and destroy the biofilm of MDR-*S. aureus*, including MRSA, when compared with linozolide and vancomycin at the same concentration. 

The destruction of biofilms triggers antibiotic re-sensitization, and the use of P5-like and short AMP in conjunction with antibiotics might be a viable approach.

### 5.4. Conventional Antibiotic Re-Sensitization 

The discovery of AMPs brings up an entirely new avenue for dealing with vancomycin-resistant *S. aureus* (VRSA). According to previous studies, peptides at sub-inhibitory concentration (sub-MIC) can resensitize VRSA to several conventional antibiotics, potentially inhibiting the development of drug resistance [143]. Concerning the re-sensitization mechanism, scientists hypothesized that sustained osmosis of peptides at sub-MIC could enhance bacterial membrane permeability and increase the likelihood that antibiotics will reach their targets. Previous investigations have shown that the human milk protein-lipoid complex HAMLET significantly re-sensitizes MRSA to methicillin and VRSA to vancomycin by depolarizing the bacterial membrane and dissipating proton dynamics [146]. Another study reported that esculentin-1b (1–18) has been shown to resensitize MDR *E. coli* to antibiotics at sub-MIC levels [147].

Nisin had a synergistic effect against MRSA when taken with penicillin, chloramphenicol, ciprofloxacin, indolicidin, or azithromycin by preventing the formation of biofilms or preventing bacterial attachment to solid surfaces [148,149]. 

The antimicrobial peptide SAAP-148 and the tetracycline antibiotic demeclocycline hydrochloride (DMCT) have been shown in trials to have a synergistic antibacterial action to treat MDR *p. aeruginosa* [150]. Additionally, Epsilon-poly-L-lysine and nisin had synergistic effects on Gram-positive foodborne bacteria *L. monocytogenes* and *B. cereus* [151].

## 6. How Were AMPs Proved to Combat Several MDR Bacteria?

### 6.1. AMPs Combat MRSA Infection

MRSA was first identified 60 years ago. Since then, MRSA infections have quickly spread around the world [152]. Cbf-K_16_, a cathelicidin-like AMP, is a mutant of bf-30 which is present in golden ring snake venom. Cbf-K_16_ was created by substituting Glu^16^ with Lys^16^ to produce more positive charges and hence boost antibacterial action [153]. Li et al. [153] studied the synergetic activity of Cbf-K_16_ when combined with ceftazidime/ampicillin to combat MRSA, which was resistant to these two antibiotics. Amazingly, the authors found that this combination significantly reduces the load of MRSA and increases the survival rate of MRSA-infected mice. Furthermore, Ib-AMP4 is a plant defense AMP derived from impatiens seed. Ib-AMP4 possesses a strong bactericidal effect against both Gram-positive and Gram-negative pathogens. Sadelaji et al. [154] investigated the bactericidal activity of Ib-AMP4 in eradicating MRSA infection. The findings of their experiment validated Ib-AMP4’s antibacterial activity against MRSA, and the results of their scanning electron microscope analysis demonstrated that Ib-AMP4 could break down MRSA’s biofilm. Additionally, Ib-AMP4 showed great therapeutic promise in the in vivo model, as all MRSA-infected animals treated with Ib-AMP4 survived, with no bacteria detected in their blood samples [154].

### 6.2. AMPs Combat ESKAPE Infections 

“ESKAPE” is derived from the first letter of multiple bacterial names: *Enterococcus faecium*, *S. aureus*, *K. pneumonia*, *A. baumannii*, *P. aeruginosa*, and *Enterobacter* spp. These bacteria are commonly referred to as superbugs and represent a significant class of highly AR bacteria that are frequently found in hospitals [17].

SAAP-148, a synthetic peptide derived from the structure of the human cathelicidin LL-37, was chosen as the most promising AMP for bacterial count reduction [155]. According to Anna et al. [156], SAAP-148 was more effective in killing bacteria under physiological settings in vitro than several other known preclinical and clinical-phase AMPs. Furthermore, they demonstrated that SAAP-148 effectively killed ESKAPE infections without causing resistance, inhibited biofilm formation, and eradicated existing biofilms and persister cells. Acute and chronic biofilm-associated infections with MRSA and MDR *A. baumannii* were totally eliminated from injured ex vivo human skin and murine skin in vivo after a single 4-h treatment with hypromellose ointment containing SAAP-148. Collectively, SAAP-148 is a potential therapeutic candidate in the fight against ESKAPE and MDR infections [156].

TC19, a synthetic peptide produced from the human thromboxane 1-derived peptide L3, is another notable example [157]. Riool et al. [158] conducted biophysical tests on the interaction of bacterial plasma membrane mimics with TC19. The findings revealed that TC19 had a strong selectivity for bacterial membranes and could efficiently and rapidly kill numerous MDR strains of ESKAPE in human plasma. Additionally, the authors showed that applying Hyproelose gel containing TC19 to superficial infected wounds in mice greatly decreased the load of MDR *A. baumannii* and MRSA.

Pseudin-2 (PSE-T2), a 24 amino acid AMP derived from frog skin, shows considerable growth-inhibitory action against Gram-negative bacteria [159]. Kang et al. [160] discovered that PSE-T2 had a substantial inhibitory impact on biofilm formation by *E. coli*, *P. aeruginosa*, *S. aureus*, and their MDR strains. The inhibition rates for *E. coli*, *P. aeruginosa*, and *S. aureus* were 89.8%, 97%, and 94.2%, respectively. Using *P. aeruginosa* as an example, their studies revealed that wounds infected with MDR *P. aeruginosa* healed considerably quicker following PSE-T2 treatment than ciprofloxacin-treated or untreated wounds.

WLBU2 is an engineered cationic peptide made up of 24 amino acids, 13 of which are arginine. WLBU2 was found to possess a high antibacterial impact on the biofilm of MDR *A. baumannii* and *K. pneumoniae* [161,162]. Surprisingly, the researchers discovered that the MIC of WLBU2 against Gram-positive and Gram-negative bacteria, including VRE and MRSA, was less than 10 μM. Moreover, the MIC of WLBU2 against extensively drug-resistant (XDR) *A. baumannii* and *K. pneumoniae* was 1.5–3.2 μM and 2.9–4.7 μM, respectively.

Additionally, Mwangi et al. [163] synthesized a cyclopeptide leader peptide ZY4 that is stabilized by disulfide bonds. This particular AMP type possesses great in vivo stability and exhibits strong action against clinical MDR strains of *P. aeruginosa* and *A. baumannii*. ZY4 has excellent plasma stability and limited drug resistance, and it can cross bacterial membranes to kill or even destroy them. The authors discovered that administering ZY4 in a mouse septicemia infection model might lower vulnerability to *P. aeruginosa* pulmonary infection as well as limit the spread of *P. aeruginosa* and *A. baumannii* to target organs. Subsequently, ZY4 is a great potential medication to treat MDR bacterial infections.

## 7. Tactics Used by Bacteria to Resist AMPs

Due to the high levels of AMPs produced locally and the widespread incidence of AMPs in the competing bacterial world, it is expected that a variety of resistance mechanisms have emerged in a variety of bacteria including *staphylococci*, oral bacteria such as *streptococci*, and enteric bacteria such as *salmonella* [164,165]. 

Most microbial species have AMP resistance genes [166], making AMP resistance mechanisms one of the most difficult barriers to using AMPs for medical purposes. Despite the great variety of bacteria that are vulnerable to AMPs, the application of such peptides as antimicrobials has indeed been challenged because certain antimicrobial peptides have wide-level cross-resistance with the aid of changes occurring in structures and function mechanisms. Passive resistance and inducible or adaptive resistance mechanisms, which are two fundamentally different tactics used for AMP resistance, have been the main topics of some investigations. Responses to environmental variables that typically manifest independently when AMPs are not present are referred to as passive resistance. The term “inducible resistance mechanisms” refers to bacterial molecular alterations brought on by the presence of AMP or the stress that it causes in both Gram-positive and Gram-negative bacteria. AMPs resistance can happen in a variety of ways, such as by altering the structure or net charge of cell walls and cytoplasmic membranes [167,168,169,170]. Table 2 summarizes the tactics used by bacteria to resist AMPs.

### 7.1. Protease-Mediated AMP Resistance

Bacteria can directly stop the action of AMPs with the peptidases and proteases that cleave AMPs at particular amino acid residue sites. By secreting proteases into the extracellular space, Gram-positive bacteria get rid of AMP activity. The majority of the proteases in Gram-negative bacteria are found on the outer membrane [171].

For instance, the metalloprotease aureolysin, which is generated by *S. aureus*, hydrolyzes the C-terminal domain of AMPs and renders peptide antibiotics like LL-37 inactive. The sarA protein suppresses and downregulates aureolysin in *S. aureus*. PgtE of *Salmonella enterica*, which encourages resistance to α-helical AMPs, is another protease that confers AMP resistance [172].

Additionally, *P. aeruginosa* creates an elastase that breaks down LL-37 in a lab setting by breaking down the peptide bonds between Asn-Leu and Asp-Phe while also promoting the survival of the bacteria [173]. 

### 7.2. Production of External AMP-Binding Molecules (Trapping)

Certain types of bacteria create proteins that are surface-anchored or released, which attach to particular AMPs with greater sensitivity and prevent them from entering the host cell’s surface and cytoplasm [173].

SIC protein, as well as several M protein serotypes produced by *Streptococcus pyogenes*, SIC, and M protein serotype 1 (M1) inactivate LL-37, so it is considered a crucial element in the invasiveness of *S. pyogenes* strains [172,174].

### 7.3. Cell Wall and Membrane Modification (Surface Remolding)

The bactericidal activity of AMPs primarily affects the cell membrane and cell wall of bacteria. To confer resistance on AMPs, certain bacteria frequently modify the components of their cell surface. These compositional variations could result in altered membrane fluidity, decreased net negative charge on the cell surface, or modifications to the molecular structure [172]. The reduction of the negative charge of the cell membrane makes the bacteria more resistant to antimicrobial peptides but is not a common tactic used by bacteria. By D-alanylates teichoic acid (TA), the negative charge of the LPS of Gram-negative bacteria is masked. Some of the microbial tactics for altering the outer surface are listed below [172,174]:

The contribution of TA to AMP resistance: AMPs function best when they have a cationic net surface charge, probably because it is simpler for them to effectively target bacterial surfaces. Teichuronic acid polymers, LPS, and phospholipids, which offer a greater cationic AMP attachment, are typical anionic substances that cover the bacterial surface. Compared to Gram-negative bacteria, Gram-positive bacteria are more resistant to AMPs because of the makeup of their various cell surfaces. As an example, they use the esterification of TA with the amino acid D-alanine to lower the net charge of either wall TA or LTA [170].

LPS modifications: adding 4-amino-4-deoxy-L-arabinose (Ara4N) to the core and lipid-A sections or adding phosphoethanolamine (PEtN), considered as a fatty acid, acetylation of the O-antigen, and hydroxylation of fatty acids are frequent mechanisms of resistance to AMPs in Gram-negative bacteria. Regulatory mechanisms such as *S. typhimurium* PhoPQ, a two-component regulatory system that collaborates with PmrAB, govern LPS alterations [175]. When LL-37 and C18G are present, the phoPQ system immediately recognizes them and triggers pmrAB to add a 4-aminoarabinose (Ara4N) molecule to the lipid-A phosphate group [174]. A larger positive charge is produced by the PmrAB regulatory mechanism, which lowers the electrostatic force interaction between antimicrobial peptides and the negatively charged phosphate of lipid A [170].

### 7.4. Capsule Production (Exopolymers)

Another layer of resistance for AMPs is provided by the capsules that bacteria release around their outer membrane. When the number of polysaccharide molecules in the capsule increases, the resistance to antimicrobial peptides also increases [169]. The genes involved in capsule biosynthesis are activated by LL-37 in *Neisseria meningitidis* to promote capsule formation. Capsule manufacturing allows *Neisseria meningitidis* to withstand human AMP LL-37 [176]. *P. aeruginosa* has also shown the capsules’ ability to neutralize AMP [177].

### 7.5. Efflux Pumps 

AMPs can also be pumped out of the cytoplasm by certain bacteria to survive them. Because they increase AMPs resistance, efflux pumps are therefore regarded as virulence factors. The effectiveness of AMPs to kill bacteria is improved by inactivating efflux pumps. Based on how they work, these pumps can be classified into two groups: primary transporters, which release AMPs through the process of ATP hydrolysis, and secondary transporters, which use transmembrane electrochemical gradients of either protons or sodium ions. This is how *Yersinia enterocolitica* resists AMPs by using the RosA/RosB efflux pump system. The RosA/RosB system blocks the formation of O-antigens or causes the cytoplasm to become more acidic because of the AMPs being pumped out by a potassium antiporter once they reach the cytoplasmic membrane. Temperature and the presence of AMPs both affect RosA/B expression. In addition, the restriction of the energy source could affect how it works. RosA is in charge of pumping AMPs into the periplasm, and RosB is in charge of controlling the pH inside the cell [169,174]. AMP resistance cannot be mediated by many other MDR exporters. MDR efflux pumps are now limited-effect resistance mechanisms as a result [167]. 

### 7.6. Biofilms 

Some bacterial biofilms’ extracellular slime matrix serves as a defense against specific AMPs [178]. The bacteria’s slimy exopolysaccharides aid in their attachment to surfaces, provide a defense against outside invaders, and deter AMPs. The polymer alginate, an acylated polysaccharide made up of anionic sugars mannuronic and glucuronic acid, which attaches to antimicrobial peptides and causes a modification in their structure due to conformational changes, is present in the *P. aeruginosa* biofilm [179]. 

The analogous intercellular polysaccharide adhesin (PIA), which is composed of poly-N-acetyl glucosamine, is what gives Gram-positive bacteria like *S. aureus* and *S. epidermidis* their resistance to LL-37. Deacetylation can boost PIA’s effect by increasing the biofilm matrix’s net positive charge. Therefore, it is still challenging to use AMPs as a therapy for diseases caused by bacteria that produce a biofilm [169,174].

## 8. How Can AMPs Win the Battle and Overcome Developed Bacterial Resistance? 

The inquiry to overcome the challenges of using AMPs as suitable therapeutic options against bacterial antibiotic resistance seems justified given their broad-spectrum activity. In comparison to traditional antibiotics, they also have a quick start to killing action and minimal levels of induced resistance. However, pathogens employ a variety of strategies to develop resistance to antimicrobial peptides; most recently, learning more about the mechanisms by which bacteria resist AMPs has aided in the identification of numerous scientific solutions. In this regard, adjustments to AMPs’ amino- or carboxyl terminal as well as changes to their molecular structure, including the addition, subtraction, and replacement of one or more amino acids, are some potential answers. 

Additionally, some researchers have proposed modifications to their biochemical characteristics, such as cationicity, hydrophobicity, and amphipathicity. 

Finally, the synergistic effects of AMPs combined with some substances, such as conventional antibiotics, can be beneficial in lowering resistance [171,180,181]. 

Using improved delivery vehicles is a key method to increase the half-life and performance of AMPs. It is interesting to note that the use of nanoparticles for AMP distribution has recently received a lot of attention because they offer a wide surface area for AMP adsorption and encapsulation as well as the ability to inhibit peptide self-aggregation [182,183,184,185,186,187,188,189]. Amazingly, a new pathogen-specific monoclonal antibody (mAb) coupled with AMP for targeted delivery was recently reported [190]. In this delivery vehicle, AMP fused to the C-terminal end of the V_H_ and/or V_L_-chain of a mAb. Johnson et al. [190] demonstrated that this novel delivery vehicle can combine the precise targeting of the bacterial surface by mAb with the potent lethal action of an AMP. The authors also showed that this delivery vehicle has low levels of toxicity to mammalian cells, kills *P. aeruginosa* strains quickly, and protects mice against lung infections caused by *P. aeruginosa*. To maximize their intracellular activities, cell-penetrating peptides (CPPs) are effective carriers that could transport a variety of payloads across biological membranes [191]. As a result, combining AMPs with CPPs might be an easy and practical way to make AMPs more bioactive. The development of CPPs as a novel strategy for combating intracellular infections is supported by mounting evidence [192]. According to a study, CPP (R9) coupled to AMPs (magainin and M15) dramatically increased antibacterial efficiency against Gram-negative bacteria [193]. 

The combination of AMPs with conventional antibiotics is another strategy to overcome bacterial resistance. This tactic involves 4 main synergistic mechanisms; increase bacterial membrane permeability; direct the potentiation of antibiotics; inhibit resistance mechanisms; and inhibit biofilm formation [18,194]. Antibiotic-peptide conjugates (APCs) are made up of a peptide and a known antibiotic joined together by a linker. The objective of this conjugate is to develop a novel, versatile antimicrobial agent with synergistic antibacterial activities while avoiding known drawbacks of antibiotics or peptides such as cytotoxicity, cellular penetration, hemolysis, serum instability, and instability in elevated salt environments [195]. The efficiency of APCs is controlled by various parameters; the selection of a particular AMP and antibiotic, including the target organism; the presence and kind of resistance mechanisms; the local microenvironment; and the concentrations and dosing schedules of the two drugs [196,197,198].

## 9. FDA-Approved AMPs and AMPs in Clinical Trials

Many AMPs were evaluated as medicinal products and received patents [199] (Table 3). Only a small subset of these AMPs has received FDA therapeutic approval. The upcoming section will discuss the AMPs in clinical trials and FDA-approved AMPs (Table 3), as well as the drawbacks of AMPs that prevented them from being approved by the FDA.

### 9.1. FDA-Approved AMPs

AMPs are produced via the ribosomal translation of mRNA as well as non-ribosomal synthesis. Non-ribosomally manufactured peptides made by bacteria, like glycopeptides and bacitracin, have been investigated as potential therapeutic agents because they resemble natural peptides. They have remarkable biological properties, ranging from antibiotics to biosurfactant functions [17,64,200]. 

Vancomycin is a non-ribosomal synthetic tricyclic glycopeptide. It consists of a 7-membered tricyclic peptide structure connected to a vancomyl-glucose disaccharide [201]. Vancomycin inhibits cell wall biosynthesis, making it particularly efficient against Gram-positive bacteria. It is used as a first-line treatment for MRSA infections, which include cellulitis, osteomyelitis, pneumonia, endocarditis, and bacteremia [202]. 

Vancomycin derivatives include oritavancin, dalbavancin, and telavancin [203,204]. They are semisynthetic lipoglycopeptides that cure infections brought on by Gram-positive bacteria which are resistant to several drugs [202]. The lipophilic side chains of the lipoglycopeptides increase the length of their half-life: oritavancin’s half-life is 195.4 h; dalbavancin’s is 14 days; and telavancin’s is 8 h. AMPs are used to treat *S. aureus*-related complex skin and skin structure infections (cSSSIs), in addition to other Gram-positive bacteria. These AMPs are anticipated to take the position of vancomycin in the treatment of cSSSIs [205]. 

Daptomycin is a 13-amino acid peptide that acts on the bacterial membrane [206]. Daptomycin and its derivative Cubicin received FDA approval in 2003 to treat *S. aureus* infections and cSSSIs. Therefore, it would seem that only Gram-positive bacteria, like MRSA and VRE, are vulnerable to its action through attachment to the cell membrane and rapid depolarization of the membrane potential. Additionally, it has a delayed post-antibiotic effect that can last up to 7 h and a long half-life of around 8 to 9 h [207].

### 9.2. The Preclinical Trial Phases of AMPs

#### 9.2.1. Phase I 

Plectasin has antibacterial, antiviral, and antifungal properties and is made up of α-helices and β-sheets. Plectasin’s unique antibacterial activity involves interaction with bacterial cell wall precursors and prevention of the synthesis of cell walls [208]. It is inefficient versus host cells such as healthy human epidermal keratinocytes, erythrocytes, and murine fibroblasts but effective versus *Strept pneumoniae*. This reveals the remarkable in vitro selectivity of the plectasin towards bacterial cells [209].

A 13-amino acid peptide called IDR-1 exhibits antimicrobial efficacy against infections of both the Gram-positive and Gram-negative types, including MRSA, VRE, and others. IDR-1 does not directly combat pathogens; instead, it regulates inflammatory responses and promotes the production of cytokines and chemokines, which are components of the innate immune system. As a result of its indirect targeting of bacteria, IDR-1 poses a low risk of developing resistance [210].

#### 9.2.2. Phase II

A synthetic 8-mer peptide called CZEN-002 is generated from α-melanocyte-stimulating hormone. Gram-positive, Gram-negative, and pathogenic fungi are all specifically targeted by CZEN-002, which also prevents the replication of the human immunodeficiency virus-1 (HIV-1) [211,212]. Phase I/II trials have already been carried out on it for the management of vulvovaginal candidiasis and the results have been promising [213].

The N-terminal of human lactoferrin is a source of the 11-mer peptide known as hLF1-11. Intravenous administration of hLF1-11 has a positive impact on animals with MRSA infection. This AMP has antibacterial and antifungal effects by activating mitochondria and generating high amounts of reactive oxygen species (ROS), thereby raising plasma membrane permeability and potential. Additionally, hLF1-11 shows effectiveness against infections linked to allogeneic bone marrow stem cell transplantation [214,215,216]. 

A synthetic antimicrobial peptidomimetic (SAMP) called LTX-109 (Lytixar) was created by Lytix Biopharma to target and destroy bacterial membranes [205]. Phase I/II clinical trials for this peptide were conducted to treat MRSA infections, while phase II clinical trials were conducted to treat skin as well as soft tissue infections, but both trials were stopped in 2015 [217].

The C-terminal of a histatin-5 amidated fragment is P113 (Demegen). Together with copper (II) and zinc (II), it forms a stable compound. It is effective against *streptococci*, *staphylococci*, *P. aeruginosa*, and *C. albicans* while binding with metals [218].

Indolicidin analogue omeganan (CLS001): It works to prevent infections brought on by *S. aureus* [219]. It is currently being tested in phase II clinical trials for patients with moderate atopic dermatitis and phase III trials for rosacea. However, phase III trials for catheter-related infections were unsuccessful [217].

#### 9.2.3. Phase III 

A 23-amino-acid amphipathic peptide known as D2A21 (Demegal) exhibits antifungal action against a variety of fungi, such as C. albicans, Aspergillus niger, and Trichophyton mentagrophytes. Additionally, it works to combat harmful germs, including P. aeruginosa and *S. aureus* [220]. A synthetic peptide with an antibacterial action against MRSA, Xoma-629 is produced from a protein that increases permeability and is bactericidal [221]. Phase II clinical trials for the treatment of impetigo with the peptide created by Xoma Ltd. were terminated after 2008. The use of this peptide in preclinical studies to treat mycoses and acne was stopped in 2006 [221].

**Table 3:** List of some FDA-approved AMPs and AMPs in clinical trials [19,217].

Peptide Name	Phase	Source	Mechanism
Friulimicin	Phase I trials	*Actinoplanes friuliensis*	Membrane disruption
NVB-302	Phase I trials	Semisynthetic a class of post-translationally modified peptides called lantibiotics	Inhibition of cell wall synthesis
Omiganan (CLS001)	Phase II trials	Synthetic analog of indolicidin	Enhancement of bacterial membrane permeability
LL-37	Phase II trials	Human cathelicidin	Membrane disruption/immunomodulation
D2A21 (Demegal)	Phase III trials	Synthetically designed peptide	Apoptosis is induced by the formation of multimeric holes in the bacterial cell wall
Gramicidin	Phase III trials	*Brevibacillus brevis*	Membrane disruption/immunomodulation
Polymyxin B	FDA-approved	*Bacillus polymyxa*	Membrane disruption/immunomodulation
Polymyxin E (Colistin)	FDA-approved	*Bacillus polymyxa*	Membrane disruption/immunomodulation
Vancomycin	FDA-approved	Glycopeptide obtained from *Streptomyces orientalis*	Inhibition of cell wall biosynthesis
Dalbavancin	FDA-approved	Teicoplanin-derived semisynthetic lipoglycopeptide	Disruption of cell wall biosynthesis
Oritavancin	FDA-approved	Chloroeremomycin-derived semisynthetic lipoglycopeptide	Suppression of trans-glycosylation and transpeptidation and breakdown of the Gram-positive bacterial cell membrane
Telavancin	FDA-approved	vancomycin-derived semisynthetic lipoglycopeptide	Interference in cell wall and peptidoglycan synthesis
Daptomycin	FDA-approved	*Streptomyces roseosporus*	Membrane interruption, and inhibition of DNA, RNA, and protein synthesis

## 10. Discovery of Novel AMPs

However, because of the negative effects mentioned above, the majority of AMPs have not been able to fully realize their therapeutic promise. The creation of an AMP Database is a substantial challenge because of the variability in the sequence and structure of AMPs. There are several methods for anticipating active AMPs using computational techniques, such as: (1) utilizing the pro-peptide and/or AMP amino acid sequences; and (2) utilizing knowledge of AMP expression and processing [217].

Additionally, novel AMP-like lantibiotics families were discovered using polypeptide-modifying enzymes; these lantibiotics may have antibacterial activity after being post-translationally modified with such enzymes. This method can identify strains that could manufacture the desired AMPs by scanning the sequenced bacterial genome for the presence of certain enzymes [222,223]. In addition, high-throughput screening techniques were developed to make it possible to quickly and precisely determine the activities of novel AMP [224]. 

**Figure 6:**
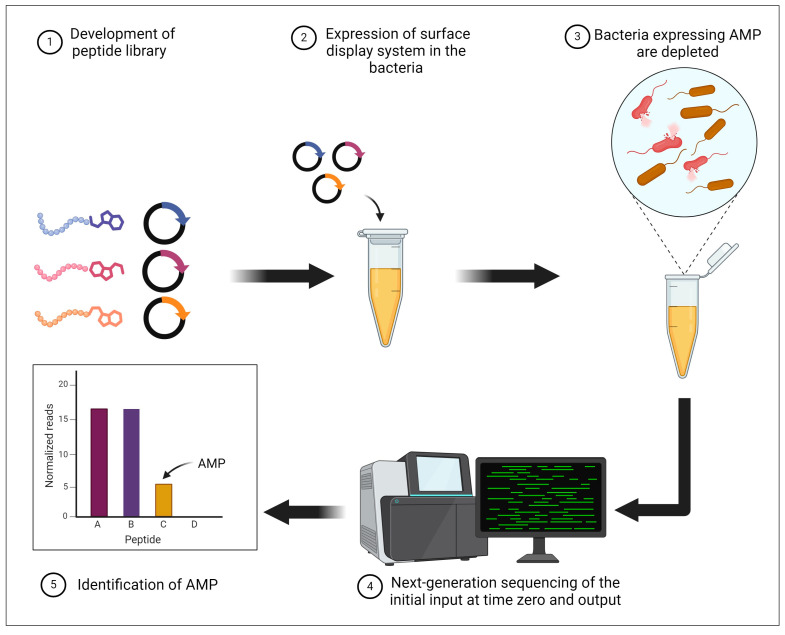
Illustration of surface localized antimicrobial display (SLAY) technology [225]. Created with BioRender.

Recently, the surface localized antimicrobial display (SLAY) technology was created [225] (Figure 6). Many unique AMPs are simultaneously generated and screened by the SLAY technique [225]. By using a structured fusion protein, this technique can first locate target peptides in the *E. coli* outer membrane. Then, the population of *E. coli* that express the fusion peptide will become less abundant when the peptides in the library have an antibiotic impact. Using this technique, scientists were able to screen new brands of AMP candidates that had not yet been found in nature. In contrast to known AMPs and their distinguishing characteristics (cationic and hydrophobic characters), this technique can identify a wide range of peptides that are not restricted by length, electric charge, or hydrophobicity. Consequently, this approach can give active AMPs a new set of qualities. Additionally, new AMP-ligand interactions can now be predicted thanks to the development of computational techniques, growing knowledge of AMP structure and function, and other factors [226].

## 11. Obstacles and Limitations Facing AMPs

Despite having a relatively good safety profile, AMPs can interact with eukaryotic cell membranes, notably those of erythrocytes, and destroy them, which can cause cytotoxicity (especially hemolysis). AMPs can attach to a receptor or operate on the membrane to cause toxicity, depending on their mode of action. The immune system is modulated for bacteriostatic action by the receptor-binding peptides. Additional inflammatory secondary diseases like rosacea, atopic dermatitis, or psoriasis can be generated by overactive or dysregulated immune responses at high AMP concentrations [227]. 

Designing novel AMPs presents difficulties in terms of identifying the structural characteristics and physicochemical traits that maximize the antibacterial activity and reduce the cytotoxicity of AMPs. The link between hydrophobicity/amphipathicity and the potential for cytotoxicity seems to be complicated, and the outcome is not dependent on a single factor but instead requires a balance between charge, hydrophobicity, and amphipathicity, which makes the theoretical prediction extremely challenging. It is well established that AMPs with higher hydrophobicity and/or amphipathicity can worsen hemolysis [122,228]. Hydrophobicity, which makes it easier for AMPs to enter bacteria membranes, is another crucial component of antimicrobial action. Nevertheless, an excessively hydrophobic peptide can also damage mammalian cells, leading to toxicity and a reduction in its selectivity for microbial cells [122]. The hydrophobic and hydrophilic residues of AMPs can be balanced to promote optimal antimicrobial activity against pathogens while limiting toxicity toward mammalian cells, which could help reduce the possible toxicity associated with excessive hydrophobicity. The selectivity of AMPs is often evaluated by therapeutic index, which is calculated as the ratio between the minimum hemolytic concentrations (MHC) and MIC, to experimentally address the danger of cytotoxicity [228].

The limited oral bioavailability of AMPs is the main barrier preventing their use in clinical settings. The amide bonds in AMPs can be successfully broken down by the same digestive enzymes that work on ingested proteins. Additionally, the strong polarity and high molecular weight of AMPs limit their intestinal permeability. The systemic delivery of AMPs is also restricted due to rapid proteolysis in the blood stream and rapid elimination from circulation by the liver and kidneys (hepatic and renal clearance, respectively), resulting in a peptide with a relatively short half-life. This lability not only allows the body to rapidly modify the amounts of peptide hormones to preserve homeostasis but also makes it challenging to develop novel therapies. Therefore, even though some AMPs are designed for oral or intravenous administration, local application is still the most prevalent method of AMP delivery. It is noteworthy that AMPs are susceptible to breakdown by tissue proteolytic enzymes even after local therapy [229,230]. A variety of techniques are used to handle the instability of AMPs at these factors to improve their stability, bioavailability, and therapeutic potential. These include methylation, C-terminal amidation, N-terminal acetylation, replacing L-amino acids with their D enantiomers, dimerization, hybridization, or encapsulating nanoparticles [231]. 

## 12. Conclusions

AMPs are promising therapeutic approaches with strong antibacterial activity against a variety of pathogens, including Gram-negative and Gram-positive bacteria, notably MDR species. This gives them a therapeutic edge over antibiotics. This is because there are few options for treating such infections. This review has provided instances of AMPs that are efficient against MDR bacteria and could potentially take the place of traditional antibiotics. The therapeutic potential of AMPs is increased by their functions to promote the activity of therapeutic molecules and the removal of biofilms. Although many AMPs have been described, few have progressed to advanced clinical trials despite the efforts made to optimize them. For AMPs to be employed in clinics, there are several obstacles to be overcome, including their low stability and cytotoxicity. However, with more research being carried out, it is anticipated that soon there will be effective antibacterial alternatives that can get rid of infections that are too dangerous to treat. By efficiently utilizing the desired properties, such as amphiphilicity and lipophilicity, modern technologies enable us to modify natural AMPs and synthesize novel AMPs. Additionally, the use of AMPs in combination with other strategies has the potential to lessen toxicity and side effects as well as stop drug resistance. In the future, we hope to create AMP-based treatment plans for bacterial infections that are safer, more targeted, and more effective.

## Figures and Tables

**Table 1:** AMPs derived from various plant sources. Modified from [80,81,82].

Plants	Peptide Name	Scope of Activity
Triticum aestivum, *Hordeum vulgare*	Plant defensins	Bacteria, oomycetes, yeast, and necrotrophic pathogens
Avocado		*E. coli*, *S. aureus*
Alfalfa		*F. graminearum*
*Raphanus sativus*		*Z. bailii*
*Potato tubers*	Snakins	Bacteria
*Pyrularia pubera*	Type II thionins (α-hordothionin and β-hordothionin)	Bacteria
*Helleborus purpurascens*	Type V thionins (Hellothionin D)	Bacteria
*Viscum album* *Phoradendron tomentosum* *Phoradendron liga*	Type III thionins; Viscotoxins, Phoratoxins, Ligatoxin	Bacteria
*Crambe abyssinica*	Type IV thionins (crambins)	Bacteria
*Poaceae*	Type I thionins (purothionins)	*Xanthomonas phaseoli*, *P. solanacearum*, and *X. campestris*, *Corynebacterium flaccumfaciens*, *Erwinia amylovora*, *C. sepedonicum*, *C. michiganense*, *C. poinsettiae*, and *C. fascians*
*Oldenlandia affinis*	Cyclotides: kalata B1 and B2	Bacteria, fungi, nematodes
*Phaseolus lunatus*	Lunatusin	Bacteria and viruses
*Phaseolus vulgaris*	Vulgarinin	Bacteria, fungi, and viruses
*Cicer arietinum*	Cicerin	Fungi and viruses
*Macadamia integrifolia*	Vicilin-like	Bacteria and fungi

**Table 2:** Tactics used by bacteria to resist AMPs.

Bacterial Tactic	Bacterial Species	Mechanism of Development
Protease-mediated AMP resistance	*S. aureus*	The metalloprotease aureolysin, which is generated by *S. aureus*, hydrolyzes the C-terminal domain of AMPs and renders peptide antibiotics like LL-37 inactive. The sarA protein suppresses and downregulates aureolysin in *S. aureus.* The sarA protein suppresses and downregulates aureolysin in *S. aureus*.
	*Salmonella enterica*	PgtE protein of *Salmonella enterica* encourages resistance to α-helical AMPs.
	*P. aeruginosa*	*P. aeruginosa* creates an elastase that breaks down LL-37 in a lab setting by breaking down the peptide bonds between Asn-Leu and Asp-Phe while also promoting the survival of the bacteria.
Production of external AMP-binding molecules (trapping)	*Streptococcus pyogenes*	SIC protein, as well as several M protein serotypes produced by *Streptococcus pyogenes*, are surface-anchored or released proteins that attach to AMPs with higher sensitivity and prevent them from entering the host cell’s surface and cytoplasm.
Cell wall and membrane modification (surface remolding)	Gram-positive bacteria	Teichoic acid (TA) modification: Gram-positive bacteria use the esterification of TA with the amino acid D-alanine to lower the net charge of either wall TA or LTA.
	Gram-negative bacteria	LPS modifications; by adding 4-amino-4-deoxy-L-arabinose (Ara4N) to the core and lipid-A sections or adding phosphoethanolamine (PEtN), acetylation of the O-antigen, and hydroxylation of fatty acids are frequent ways of resistance to AMPs.
Capsule production	*Neisseria meningitidis*	The genes involved in capsule biosynthesis are activated by LL-37 in *Neisseria meningitidis* to promote capsule formation. Capsule manufacturing allows *Neisseria meningitidis* to withstand human AMP.
	*P. aeruginosa*	*P. aeruginosa* can produce the capsules to neutralize AMPs and increase the resistance.
Efflux pumps	*Yersinia enterocolitica*	*Yersinia enterocolitica* resists AMPs by using the RosA/RosB efflux pump system. The RosA/RosB system blocks the formation of O-antigens or causes the cytoplasm to become more acidic because of the AMPs being pumped out by a potassium antiporter once they reach the cytoplasmic membrane.
Biofilm formation	*P. aeruginosa*	In *P. aeruginosa* biofilm, the polymer alginate, an acylated polysaccharide made up of anionic sugars mannuronic and glucuronic acid, attaches to antimicrobial peptides and causes a modification in their structure due to conformational changes.
	*S. aureus* and *S. epidermidis*	The intercellular polysaccharide adhesin (PIA), which is composed of poly-N-acetyl glucosamine, is responsible for *S. aureus* and *S. epidermidis* resistance to LL-37. Deacetylation can boost PIA’s effect by increasing the biofilm matrix’s net positive charge.
